# Retraction: Comparison of Challenges and Problems Encountered in the Practice of Exclusive Breast Feeding by Primiparous and Multiparous Women in Rural Areas of Sindh, Pakistan: A Cross-Sectional Study

**DOI:** 10.7759/cureus.r48

**Published:** 2022-03-17

**Authors:** Sana Zafar, Khizer Shamim, Syeda Mehwish, Mohsin Arshad, Rahil Barkat

**Affiliations:** 1 Biological Sciences, Victoria University of Wellington, School of Biological Sciences, Wellington, NZL; 2 Medicine, Ziauddin Hospital, Karachi, PAK; 3 Dentistry, Karachi Medical and Dental College, Karachi, PAK; 4 Department of Internal Medicine, Multan Medical and Dental College, Karachi, PAK; 5 Indus Hospital Research Center, The Indus Hospital, Karachi, PAK

This article has been retracted due to the unknown origin of the data, lack of verified IRB approval, and purchased authorships. The primary author, Rahil Barkat, was involved in data theft and misuse in two recently published Cureus articles, which have since been retracted.

As the origin of this article’s data and verified IRB approval cannot be confirmed, we have made the decision to retract this article. Cureus has confirmed that the co-authors were asked by Mr. Barkat to proofread the article and provide payment in exchange for authorship. (Proofreading is an insufficient contribution to warrant authorship as defined by ICMJE.) These payments were made in the guise of “editing fees” but greatly exceed any editing fees paid to Cureus. While these authors may have been defrauded by Mr. Barkat, they remain complicit due to their lack of honest contributions to the article.

